# The Performance of Protein Meal from *Hermetia illucens* Larvae in Hetero-Clarias Hybrid Farming

**DOI:** 10.3390/insects16121279

**Published:** 2025-12-17

**Authors:** Bogdan Georgescu, Carmen Georgescu, Tudor Păpuc, Marius Vasiu, Dănuț Struți, Anca Boaru

**Affiliations:** 1 Department of Zoology and Ecology, Faculty of Animal Science and Biotechnologies, University of Agricultural Sciences and Veterinary Medicine Cluj-Napoca, 400372 Cluj-Napoca, Romania; bogdan.georgescu@usamvcluj.ro; 2 Endocrinology Clinic, Cluj County Emergency Clinical Hospital, 400347 Cluj-Napoca, Romania; cgeorgescu@umfcluj.ro; 3 Department of Endocrinology, Faculty of Medicine, “Iuliu Haţieganu” University of Medicine and Pharmacy, 400012 Cluj-Napoca, Romania; 4 Department of Aquaculture, Faculty of Animal Science and Biotechnologies, University of Agricultural Sciences and Veterinary Medicine Cluj-Napoca, 400372 Cluj-Napoca, Romania; tudor.papuc@usamvcluj.ro; 5 Doctoral School of Engineering Agricultural Sciences, University of Agricultural Sciences and Veterinary Medicine Cluj-Napoca, 400372 Cluj-Napoca, Romania; 6 SC Artema SRL, 440030 Satu-Mare, Romania; danut.ioan2193@gmail.com

**Keywords:** *Hermetia illucens*, hetero-clarias hybrid, alternative protein, enzyme complex, growth parameters

## Abstract

In the context of healthy and sustainable alternatives to fishmeal, insect meal asserts itself as a potentially healthy ingredient in aquafeeds. The aim of this study was to determine the possibility of successfully replacing fishmeal with *Hermetia illucens* larvae meal in the diet of the hetero-clarias hybrid, and to improve the overall bioavailability of the meal by supplementation with an enzyme complex (Hostazyme X). There were eight treatments: 0, 40, 50, and 60% replacement of fishmeal with larvae meal in the diet, with and without the enzyme supplement. In total, 240 fish with a mean weight of 11.43 ± 0.32 g were employed in the treatments for 80 days. Growth parameters (specific growth rate, feed conversion ratio, daily feed intake, daily weight gain, percentage weight gain, survival rate, production index, metabolic grow rate), hematological parameters and blood serum biochemistry were determined and analyzed. There was an improvement (*p* < 0.05) in most growth parameters for fish fed experimental diets. Blood parameters, although significantly different (*p* < 0.05) in some cases, were within a normal range for fish physiology. Thus, the partial replacement (40%) of fishmeal with larvae meal and supplementation with an enzyme complex produced the best growth performance compared to other treatments and controls.

## Simple Summary

In this study, we tested whether fishmeal, a commonly used ingredient in aquafeeds, can be partly replaced with black soldier fly (*Hermetia illucens*) larvae meal. We have also checked if adding a commercial enzyme complex (Hostazyme X) would help fish absorb this insect-based feed better. The experiment involved 240 hybrid catfish (hetero-clarias) fed different diets for 80 days: some with only fishmeal, and others where 40%, 50%, or 60% of the fishmeal was replaced with larvae meal, with or without enzymes. The fish fed the insect-based diets generally showed better growth, and their blood parameter values, while sometimes different, remained within healthy limits. The best results came from the diet where 40% of fishmeal was replaced with larvae meal and combined with the enzyme supplement. This suggests that insect meal, especially with enzymes, can successfully replace part of fishmeal in the aquaculture of hetero-clarias.

## 1. Introduction

Currently, the aquaculture industry is experiencing accelerated development due, on the one hand, to the growing demand for fish products and by-products and, on the other hand, to technological progress in breeding technologies. The competition for food exerts increasing pressure on the aquatic environment, while the fish reserves of the seas and oceans, as well as of the continental waters, are increasingly limited. Fishmeal and fish oil are considered the most nutritious and digestible ingredients for fish, but their use in aquafeeds has experienced a steady downward trend in recent years [1]. This is due to the decreasing quantities of ocean fish caught and the consequent increase in prices [1]. However, the demand from the aquaculture feed production industry is increasing [2]. By 2030, the majority of fish production will enter human consumption and only 10% will be used to obtain fishmeal and fish oil [3].

The valuable nutritional properties of fish oil and fishmeal for human and farm animal nutrition are well known. In feeds intended for farmed fish, it is necessary to use fishmeal (crude protein over 72%) and fish oil, due to the balanced structure in amino acids and the high nutritional value, which make it possible to properly balance combined feed recipes [4,5,6,7].

In this context, numerous studies in the field of farm animal nutrition are focused on the identification of alternative sources of protein that can replace fishmeal under economic efficiency. Thus, insect meal has been shown to have a similar nutrient profile to that of fishmeal. Due to the high content of proteins, lipids, essential amino acids, vitamins and minerals, it is a promising alternative to fishmeal [8,9]. Among candidate insects as potential alternative sources to fishmeal, the species *Hermetia illucens* presents interest due to the valuable meal that can be obtained relatively easily and at low cost. It is characterized by a high level of protein (35–50%) and fat (20–35%), which makes it a valuable source of nutrients for aquatic species [10,11,12]. The proportion and concentration of these components in *H. illucens* larvae is strongly influenced by the growth substrate, thus explaining the large limits in which these components can be found in the meal [13,14]. A series of studies demonstrate the feasibility of using *H. illucens* protein in the food of most farm animals or pets, including in the food of other insect species [15,16,17,18]. Current research shows that fishmeal can be successfully substituted with *H. illucens* meal in proportions dependent on species and age category. Thus, in feed intended for rainbow trout (*Onchorhynchus mykiss*), prepupe meal can replace up to 25% fishmeal without affecting weight gain or feed conversion index [19]. For the same species, Stamer et al. [20] conclude that meal obtained from prepupae raised on plant residues can replace fishmeal in the feed of rainbow trout in a proportion of up to 50%.

The production of the hetero-clarias hybrid (*Heterobranchus longifilis* ♂ × *Clarias gariepinus* ♀) is of economic interest due to its very good growth performance, superior feed conversion rates, high growth density in recirculating aquaculture systems, increased resistance to diseases, and tolerance to diverse water parameters [21]. Fawole et al. [22] and Adeoye et al. [23] demonstrated that *H. illucens* meal can effectively replace 50% fishmeal in feed for African catfish (*Clarias gariepinus*), while maintaining good production performance and adequate stock health status. Bartucz et al. [24] showed that *H. illucens* meal can be used with success in feeds for brood African catfish and as a feed in fry stages. Improved growth for hetero-clarias was also observed by Bake et al. [25] when using different insect meals. This is in line with previous research confirming the potential of *H. illucens* for the hetero-clarias hybrid diets [26]. However, it should be mentioned that meal from *Hermetia* larvae has a content of 3.85% chitin [27], a hard-to-digest polysaccharide that can affect the degree of utilization of nutrients in the feed. Interestingly, in African catfish, chitinase activity has been identified in both the stomach and intestine [28,29,30]. However, according to Rapatsa et al. [28], this activity appears to be insufficient for effective chitin digestion. Consequently, the lack of an efficient endogenous enzymatic system capable of degrading this polysaccharide leads to an anti-nutritional effect that directly impairs nutrient utilization from the feed [31]. The digestibility of other components, such as fiber or plant-derived carbohydrates, also presents a problem that can be solved in aquaculture by various enzyme supplements [32].

The aim of the study is to determine the possibility to successfully replace fishmeal with *H. illucens* larvae meal in the diet of the hetero-clarias hybrid, and also to improve the overall bioavailability of the larvae meal by supplementation with an enzyme complex (Hostazyme X).

## 2. Materials and Methods

### 2.1. Diet Preparation and Experimental Design

The feeding trial was conducted at the Aquaculture Laboratory within the Faculty of Animal Science and Biotechnologies, UASVM Cluj-Napoca, in 2022. The biological material consisted of the hetero-clarias hybrid.

The *H. illucens* larvae meal was produced in the aforementioned laboratory. The other ingredients used in the formulation of the feeds were purchased from commercial producers. The *H. illucens* larvae were reared on a vegetable substrate (Gainesweille diet) [33] and larval meal was produced by drying at 40 °C in a ventilated oven and subsequent grinding. The chemical composition of the meal from *H. illucens* larvae was the following: dry matter (DM%)—91.7%; crude protein—50.13%; crude fat—27.24%; crude fiber—8.7%; ash—10.7; metabolizable energy—4910 kcal/kg. Feeds were granulated with a commercial granulator at 60 °C. The obtained pellets had a diameter of 1.5 mm.

The 8 diets tested were iso-proteic and iso-energetic, being formulated according to the nutritional requirements for juveniles of the species *C. gariepinus*, according to NRC [34] (Table 1). The fishmeal ingredient was progressively substituted in the experimental diets in percentages of 40, 50 and 60% with *H. illucens* meal, respecting the same iso-proteic and iso-energetic balance (Table 1). To evaluate the effect of the enzyme supplement, each diet was duplicated and supplemented at 0.02% with the Hostazyme X complex, obtained following a dry fermentation process, by using the species *Trichoderma longibrachiatum*, composed of endo 1,4 β-xylanases, endo 1,3(4) β-glucanases, proteases, α-amylases, galactosidases, cellulases, and hemicellulases. The specific ingredients of each batch were weighed according to the proportion of inclusion in the feed, homogenized and granulated to 1.5 mm, dried, tightly packed, appropriately labeled and frozen at −20 °C until use.

The fingerlings of hetero-clarias used for the experiment were purchased from a local farm and were acclimated into the experimental rearing system for three weeks before the start of the feeding trial. A total number of 240 fish with a mean weight of 11.43 ± 0.32 g were randomly allocated to eight (8) distinct groups, in triplicate, with each replicate tank containing 10 fish. The feeding trial lasted 80 days. Twenty-four (24) rectangular plastic tanks (80 × 58 × 44 cm, with a working volume of 150 L) were used for the feeding trial. Each tank was filled with water and connected to an air pump (type CHJ-2500 Eco, output 2500 L/h, 45 W) to ensure adequate aeration. A recirculating system was employed, with one commercial canister filter for each tank. The fish were hand-fed to apparent satiation three times a day (at 8:00, 12:00 h and 16:00 h). Feed consumption was estimated by quantifying the feed administered each time and subtracting the estimated feed quantity remaining on the bottom of the tank, removed after each feeding. The water quality parameters were monitored daily with a thermometer and a colorimetric kit (JBLProAqua Test Combi Set Plus, JBL GmbH & Co. KG, Neuhofen, Germany). The following values for water parameters were maintained: temperature 28.61 ± 0.01 °C; pH 7.5 ± 0.15; dissolved oxygen 5.5 ± 0.09 mg L^−1^; ammonia (NH_3_) 0.18 ± 0.03 mg/L; nitrites (NO_2_) 0.04 ± 0.01 mg/L; nitrates (NO_3_) 0.08 ± 0.02 mg/L. The fish were maintained under a natural photoperiod. Mortalities were noted and removed daily.

### 2.2. Productive Performance Evaluations

The final weight was determined by weighing. The formulas below were used for the evaluation of growth performance, feed efficiency and nutrient utilization, according to Fawole et al. [22], Adeoye et al. [23,35] and Bain [36]:

Daily feed intake (DFI g/fish/day) = [total feed consumption (g)]/[feeding period (days) × number of fish harvested]

Daily weight gain (DWG, g/day) = (final weight − initial weight)/days

Feed conversion ratio (FCR) = [feed consumption (g)]/[body weight gain (g)]

Survival Rate (SR) = (total number of fish harvested)/(total number of fish stocked) × 100 (%)

Production index (PI) = [survival rate × (final weight − initial weight)]/(rearing period days)

Specific growth rate (SGR%/day) = [ln final body weight (g) − ln initial body weight(g)]/(number of feeding days) × 100

Metabolic grow rate (MGR g × kg^−0.8^ day^−1^) = [net weight gain (g)]/{[(initial body weight in g ÷1000)^0.8^ + (final body weight in g ÷1000)^0.8^] ÷ 2} ÷ feeding days

Percentage Weight Gain (WG%) = [final body weight (g) − initial body weight(g)]/[initial body weight (g)] × 100

### 2.3. Sampling Procedure

At the end of the feeding trial, after an accommodation period of 24 h without feed, the fish were individually weighed (digital balance electronic Kern EHA 500-2, accuracy 0.01 g, KERN & Sohn GmbH, Balingen, Germany) according to each replicate and group for the determination of final body weight, which was used for the determinations of the other growth performance indices. Blood samples were collected from a number of 2 fish randomly selected from each replicate (*n* = 6 fish/group), anesthetized previously with 100 mg L^−1^ clove oil [35]. The blood sample was collected from the fish via the caudal vein using a 2 mL hypodermic syringe and partially transferred into a tube with lithium heparin anticoagulant for the hematological investigation and partially transferred in a CLOT vacutainer for serum biochemistry analyses. The extracted blood volume from each fish was 2 mL. The blood samples were immediately stored in a coolbox (4 °C) and transported to the Hematology Laboratory, Veterinary Medicine Faculty, UASVM Cluj-Napoca. The hemoglobin and biochemical parameters were determined according to standard methods [36]. The transportation time was under 20 min after collection. The 2 fish selected for blood sampling per replicate were further euthanized [37,38] (600 mg L^−1^ clove oil, 10 min exposure time) and sampled for the chemical composition of meat.

### 2.4. Hematological Analysis

Spectrophotometric techniques were used for the hematological profile with wavelength (λ) readings specific to each monitored parameter. Thus, within the hematological profile, the values for hemoglobin (Hb, g/dL, λ = 546 nm), hematocrit (Hct, %, centrifugation at 12,000 rpm) and erythrocytes (Ery, mil/mm^3^, λ = 546 nm) were recorded. The AMP diagnostic kit was used for hematocrit determination. Glutathione peroxidase (GPx, U/g Hb) and superoxide dismutase (SOD, U/g Hb) were determined from total blood, following the Diazyme USA commercial kit (Diazyme Laboratories, Inc., Poway, CA, USA).

### 2.5. Biochemical Analysis of Serum

The biochemical parameters of the serum were determined according to standard methods [37,38]. The Screen Master Touch UV-VIS analyzer (Hospitex Diagnostics, Florence, Italy) with a commercial kit was used and the samples were centrifuged at 5000 rpm (for 5 min) in a Hettich Rotofix 32 centrifuge (Andreas Hettich GmbH & Co. KG, Tuttlingen, Germany). The AMP diagnostic kit was used for the determination of serum biochemistry parameters. The determination of total protein (g/dL), albumin (g/dL), urea (mg/dL), creatinine (mg/dL), γ-globulin (g/dL), cholesterol (mg/dL), triglycerides (mg/dL), total lipids (mg/dL), and aspartate aminotransferase (ASAT U/L), alanine aminotransferase (ALAT U/L) was conducted using the mentioned commercial diagnostic kit and according to the laboratory protocol (Hematology Laboratory, Veterinary Medicine Faculty, UASVM Cluj-Napoca).

### 2.6. Proximate Body Composition

The proximate composition was determined for the fillet and analyzed as per the AOAC procedure [39,40]. Dry matter was determined by oven drying, under constant temperature (105 °C) and airflow conditions. Crude protein was determined by the Kjeldahl method. Crude fat was determined by the Soxhlet method. Ash was determined through the high-temperature combustion of the organic matter.

### 2.7. Statistical Analyses

All data were analyzed using GLM procedures ver. 10.0 (StatSoft Inc., 2011, Tulsa, OK, USA). The experiment had a 4 × 2 completely randomized factorial design. Data sets were verified for normal distribution with the Shapiro–Wilk test and for homogeneity with the Levene test. A two-way ANOVA test was performed to evaluate the main effects of the level of inclusion of insect meal in the diets (40, 50 and 60%) compared to a standard diet, without and with enzyme supplement (−/+), as well as the interaction of these factors (level of meal and enzymes). As all data was normally distributed and variances were homogenous, only ANOVA was further used. Differences were considered significant when *p* < 0.05. The post hoc test employed was Tukey’s HSD test, to determine between which data sets there were significant differences. All data were presented as mean ± standard error.

## 3. Results

The administration of *H. illucens* insect meal in different proportions as a substitute for fishmeal in the feed of hetero-clarias and the supplementation of the feeds with exogenous enzymes influenced (*p* < 0.05) the final body mass of the fish, the average DWG (g/day) and the FCR (Table 2). In addition, the fish SR was not affected by the treatments applied in the diet (*p* > 0.05) (Table 2).

The use of *H. illucens* larvae meal in the feed of hetero-clarias juveniles improved the body mass of the fish compared to the control group (Table 2). This occurred even when the fishmeal in the feed was substituted by 60%. The use of exogenous enzymes in the feed with insect meal led to an increase (*p* < 0.05) in the body mass of the fish, obtained at the end of the experiment. In addition, the substitution of fishmeal in the feed with insect meal in a proportion of 40% and the addition of enzymes (L40+) allowed us to obtain the best body mass among the treatments.

Regarding DFI, no differences were observed among the treatments (Table 2). For DWG, the best results were observed in the LE40+ and LE50+ groups compared with the other treatments (Table 2).

The use of insect meal in fish feed led to the improvement of the FCR, the best degree of feed utilization being recorded by the group in which the fish meal was replaced by insect meal in a proportion of 40% (*p* < 0.05) (Table 2). When the enzymes were added to the feed, the tendency to improve the FCR increased, and, compared to the control groups, the FCR in L40+, L50+ and LC60+ was significantly better (*p* < 0.05).

When the insect meal was included in the fish feed, the value of PI increased in the experimental groups with enzyme supplemented diets, even when the fishmeal in the feed was replaced up to 60%. In the case of diets without enzyme supplementation, only the PI from the LE40− group increased (*p* < 0.05) (Table 3). The subsequent addition of enzymes in the combined feeds containing insect meal led to an increase in the PI value. Compared to the control groups, the PI value associated with the LE40+ group was significantly (*p* < 0.05) higher. However, in all groups, a tendency of the PI value to decrease (*p* > 0.05) was observed with the increase in the proportion of fishmeal substitution with insect meal in the feed (Table 3).

SGR was better (*p* < 0.05) in all treatments with enzymes and in LE40− compared to both controls. Compared to control groups, MGR was significantly (*p* < 0.05) better in LE40−, LE40+ and LE50+ groups. WG had significantly higher values (*p* < 0.05) compared to the control groups in LE40−, LE40+ and LE50+ (Table 3).

Blood biochemical indices that indicate a normal health status of the fish, as hemoglobin (g/dL), hematocrit (%) and erythrocytes (mil/mm^3^) did not show different values (*p* > 0.05) among the groups (Table 4).

The use of insect meal and exogenous enzymes in the feed of hetero-clarias influenced (*p* < 0.05) the blood biochemical parameters associated with the enzyme, protein and lipid profiles (Table 4 and Table 5). The highest value for the hepatic indicator GPx was observed in LE60−, significantly higher (*p* < 0.05) than in LE40−, LC+ and LE40+. The inclusion of *H. illucens* meal in the fish diet shows a decrease in the SOD values of the LE40− and LE40+ groups, and an increase in the LE60− groups (Table 4).

The highest ALAT and ASAT activities (*p* < 0.05) were observed in the LC60− and LC60+ treatments compared to the other groups (Table 4).

Serum total proteins decreased when insect meal was included in the fish feed (*p* < 0.05) compared to the value in LC−, and the addition of enzymes to the feed led to a further decrease (*p* < 0.05) (Table 5).

The albumin levels increased significantly (*p* < 0.05) in LE50− and LE60− compared to LC−, and in LE50+ and LE60+ compared to LC+. The γ-globulin levels did not change significantly (*p* > 0.05). Protein metabolism products, urea (mg/dL) and creatinine (mg/dL) were influenced by the treatments applied in the feed (Table 5). The serum urea level decreased significantly (*p* < 0.05) in LE40− compared to LC− (1.28 vs. 1.69 mg/dL). LE40+ presented a significantly lower level (*p* < 0.05) of urea compared to all other groups (Table 5). Creatinine levels decreased significantly (*p* < 0.05) in all groups with enzyme supplements compared to those without enzyme supplements.

Cholesterol increased (*p* < 0.05) in LE50− and LE60− compared to LE40−, and showed lower values in LE40+ compared to LC+, LC−, LE40−, LE50−, LE60− and LE60+. The highest level of triglycerides was observed in LE60−, significantly higher (*p* < 0.05) than in LC−, LE40−, LC+, LE40+ and LE50+. Total lipids increased in LE60− compared to LC−, LE40−, LC+, LE40+ and LE50+ (Table 5).

The use of insect meal and exogenous enzymes in the feed of hetero-clarias did not influence (*p* > 0.05) the dry matter and the crude protein content of the meat (Table 6). The level of crude fat in fish meat from groups LE50− and LE60− increased (*p* < 0.05) compared to LC−. The level of ash was lower (*p* < 0.05) in LE50− compared to LE60−, while there were no significant differences observed among the groups with enzyme supplements.

## 4. Discussion

The study demonstrates the possibility to successfully substitute fishmeal in different proportions in the diet of hetero-clarias with insect meal obtained from *H. illucens* larvae. It also highlights, for the first time for this species, the importance of using the enzyme supplement in the feed for a more efficient utilization of the insect meal. It should be emphasized that there was no case of food refusal in the experimental groups, regardless of the level of fishmeal substitution with insect meal, confirming the findings of Fawole et al. [22] under similar experimental conditions.

The gradual increase in the degree of substitution of fishmeal with meal from *H. illucens* larvae in the experimental groups was associated with a progressive increase in crude fat intake and, consequently, in metabolizable energy. Thus, most of the experimental groups presented better values compared to the control groups for the growth parameters studied. A similar experiment did not obtain significant differences (*p* > 0.05) compared to the control, at a 50% fishmeal substitution rate [23]. A possible explanation lies in the differences in the experimental design, where the fish had a lower initial weight and the experimental period was 6 weeks [23]. The FCR was significantly better in all experimental groups compared to the controls (*p* < 0.05), the most effective conversion rate being recorded in LE40+. The reduction in the conversion values could be explained by the bioavailability of larvae meal and by the particularities of the digestive system of hetero-clarias, which could overcompensate the anti-nutritional effects of chitin, demonstrating the efficient use of larvae meal by the hybrid. A similar experiment on hetero-clarias weighing 200 ± 25 g on average conducted for 6 weeks recorded the best FCR value (1.4) at a substitution rate of 50% fishmeal with *H. illucens* larvae meal, but the difference was not significant compared to other treatments or control [41]. Other studies on *C. gariepinus* showed a significantly better FCR (1.48) at a 50% replacement of fishmeal with *H. illucens* meal, in a 60-day trial [22]. Most studies on *C. gariepinus* juveniles confirm the better FCR in groups where fishmeal was partially replaced by *H. illucens* larvae meal [42,43]; however, this study is one of the first to highlight the significantly better FCR values for the hetero-clarias hybrid juveniles fed with diets where fishmeal was partially replaced with *H. illucens* larvae meal.

Regarding the SGR, in LE40− and in the case of all groups where the enzyme supplement was used except for LC+, significant differences were obtained (*p* < 0.05), which shows that a substitution of 40% fishmeal is an appropriate level, and the enzyme supplement improves the utilization of insect meal. Thus, the enzymes further increased the bioavailability of nutrients from the larvae meal [33]. Bonomini et al. [44] noted that, in an *H. illucens* protein digestibility test, the presence of chitin prevents protein digestion, with the fraction rich in chitin registering the lowest amount of solubilized protein. It is possible that the hetero-clarias hybrid has chitanase activity that may improve feed utilization, as the enzyme complex used did not have chitanase activity, and was used for overall feed utilization improvement. This can be seen from the values of some growth parameters obtained, such as SGR and FCR, which suggest a species-specific capacity to at least partially overcome the antinutritional effects of chitin.

Usually, an alteration of the hematological profile, namely a decrease in hematocrit, hemoglobin and erythrocytes, is associated with nutritional deficiency, as shown by Tacon [45], or with exposure to other environmental stressors [46]. In our study, hematological parameters had relatively constant values in all groups, with no significant differences being recorded between groups (*p* > 0.05). The values of the hematological parameters obtained in the experiment were within the limits reported for the same species or close species [46,47]. This demonstrates that the protein from *H. illucens* larvae matches the metabolic profile of hetero-clarias at a fishmeal substitution rate of up to 60%, with or without the enzymatic supplement used.

The lowest serum urea concentration observed in LE40+ indicates reduced protein catabolism and/or efficient nitrogen utilization compared to LC-, which may reflect improved dietary protein retention or suppressed amino acid deamination [48,49]. However, the value remained within the normal physiological range for teleost fish and does not suggest impaired renal function [50]. In contrast, the higher urea levels recorded in LC− and LE60− suggest relatively greater nitrogen turnover, which is consistent with increased protein metabolism rather than pathological dysfunction. Overall, the pattern of variation indicates that the experimental treatments significantly modulated nitrogen metabolism without inducing uremic stress. The lowest values of SOD were also recorded in these groups. The lowest SOD activity observed in LE40+ indicates a reduced superoxide-scavenging capacity compared to LC−, which could suggest a weakening of the antioxidant defense at this inclusion level. However, this value remained within the normal physiological range for teleost fish and does not indicate pathological oxidative stress alone [51]. The increase in the intake of *H. illucens* meal, with and without the enzyme supplement, is also accompanied by the simultaneous increase in the intake of fat and chitin from the ration, a fact materialized by the significant increase (*p* < 0.05) in the values of ASAT and ALAT in LE60+, LE60− and LE50−. The progressive increase in ALAT and ASAT values with increasing levels of insect fat in the ration suggests an overload of the liver function. The moderate increase in transaminases correlates most frequently with the deposition of fat in the liver, or with exposure to some chemical substances [52]. Regarding cholesterol, it should be emphasized that the values obtained were within the limits reported by Okorie-Kanu & Unakalamba [50].

Regarding the chemical composition of the meat of the hetero-clarias hybrid, there were statistically significant differences (*p* < 0.05) only in terms of the crude fat content in groups LE60−, LE50−, and LE60+ compared to the control groups (LC− and LC+). In the current study, this could have occurred due to the slightly higher fat percentage in the diets with a higher rate of larvae meal inclusion (Table 1). A similar experiment obtained significant differences in crude protein, but the values recorded for crude fat were not statistically significant [22].

## 5. Conclusions

The best production parameters for the hetero-clarias hybrid juveniles were obtained in the treatment where fishmeal was replaced with *Hermetia illucens* larvae meal in a proportion of 40% in the diet. Supplementing the diets with the enzyme complex Hostazyme X improved some of the productive parameters, allowing a replacement rate of up to 60% of fishmeal with *H. illucens* larvae meal.

## Figures and Tables

**Table 1:** Experimental diets and nutritive value of the feed, (+)-feed with added enzymes.

Specification	Experimental Groups							
	LC−	LC+	LE40−	LE40+	LE50−	LE50+	LE60−	LE60+
Experimental diets (% in feed)								
Wheat gluten	25.0	25.0	26.0	26.0	26.6	26.6	27.1	27.1
Starch	24.0	24.0	23.0	23.0	22.4	22.4	21.9	21.9
Soybean meal	22.0	22.0	22.0	22.0	22.0	22.0	22.0	22.0
Fishmeal	12.0	12.0	7.2	7.2	6.0	6.0	4.8	4.8
Fish oil	11.0	11.0	11.0	11.0	11.0	11.0	11.0	11.0
Blood meal	5.0	5.0	5.0	5.0	5.0	5.0	5.0	5.0
*H. illucens* meal	-	-	4.8	4.8	6.0	6.0	7.2	7.2
Premix vit-min	1.0	1.0	1.0	1.0	1.0	1.0	1.0	1.0
Enzymes	-	0.02	-	0.02	-	0.02	-	0.02
Nutritive value								
ME (kcal/kg) *	4350.20	4350.20	4414.20	4414.20	4431.60	4431.60	4448.60	4448.60
Moisture (%)	8.6	8.6	8.05	8.05	7.73	7.73	7.34	7.34
Crude protein (%)	43.90	43.90	43.73	43.73	43.96	43.96	44.12	44.12
Crude fat (%)	2.00	2.00	2.84	2.84	3.05	3.05	3.26	3.26
Fiber (%)	1.01	1.01	1.43	1.43	1.54	1.54	1.65	1.65
Ash (%)	2.81	2.81	2.84	2.84	2.85	2.85	2.86	2.86

*—calculated values from the Hepher formula: ME^DM^ (kcal/kg) = 10⋅(5.6⋅CP_DM_ + 9.0⋅Fat_DM_ + 4.1⋅NFE_DM_); ME—metabolizable energy; LC−—control group without enzyme complex; LE40−—experimental group where 40% of fishmeal was substituted with larval meal, without enzyme complex; LE50−—experimental group where 50% of fishmeal was substituted with larval meal, without enzyme complex; LE60−—experimental group where 60% of fishmeal was substituted with larval meal, without enzyme complex; LC+—control group with enzyme complex; LE40+—experimental group where 40% of fishmeal was substituted with larval meal, with enzyme complex; LE50+—experimental group where 50% of fishmeal was substituted with larval meal, with enzyme complex; LE60+—experimental group where 60% of fishmeal was substituted with larval meal, with enzyme complex.

**Table 2:** Effects of feeding *Hermetia illucens* insect meal and addition of exogenous enzymes to hetero-clarias on productive performance.

Specification	Initial Weight	Final Weight	DFI (g/ind/day)	DWG (g/day)	FCR	SR%
Larval meal effect						
LC−	11.35 ± 0.32 ^a^	27.39 ± 1.07 ^a^	0.39 ± 0.03 ^a^	0.19 ± 0.01 ^a^	2.02 ± 0.16 ^a^	96.67 ± 3.33 ^a^
LE40−	11.19 ± 0.34 ^a^	30.85 ± 1.25 ^b^	0.40 ± 0.03 ^a^	0.24 ± 0.01 ^b^	1.65 ± 0.13 ^b^	100 ^a^
LE50−	11.39 ± 0.31 ^a^	29.26 ± 1.14 ^b^	0.38 ± 0.03 ^a^	0.22 ± 0.02 ^ab^	1.81 ± 0.20 ^c^	100 ^a^
LE60−	11.17 ± 0.33 ^a^	29.19 ± 1.18 ^b^	0.38 ± 0.03 ^a^	0.22 ± 0.01 ^ab^	1.77 ± 0.18 ^c^	100 ^a^
Enzyme effect						
LC+	11.43 ± 0.25 ^a^	26.9 ± 1.15 ^a^	0.39 ± 0.04 ^a^	0.20 ± 0.02 ^a^	2.07 ± 0.21 ^a^	96.67 ± 3.33 ^a^
LE40+	11.75 ± 0.38 ^a^	34.09 ± 1.49 ^c^	0.43 ± 0.03 ^a^	0.30 ± 0.02 ^c^	1.44 ± 0.10 ^d^	96.67 ± 3.33 ^a^
LE50+	11.73 ± 0.29 ^a^	32.45 ± 1.08 ^d^	0.42 ± 0.03 ^a^	0.26 ± 0.02 ^d^	1.62 ± 0.11 ^b^	100 ^a^
LE60+	11.44 ± 0.31 ^a^	31.55 ± 1.14 ^d^	0.42 ± 0.03 ^a^	0.25 ± 0.01 ^b^	1.68 ± 0.15 ^b^	100 ^a^
*p*-value						
Meal effect	0.820	0.001	0.199	0.001	0.001	0.158
Enzyme effect	0.523	0.012	0.103	0.001	0.049	0.912
Meal × enzyme	0.524	0.334	0.561	0.001	0.043	0.794

Different superscripts in the same column show significant differences (*p* < 0.05); DFI—average daily feed intake; DWG—average daily weight gain; FCR—feed conversion ratio; SR%—survival rate; LC−—control group without enzyme complex; LE40−—experimental group where 40% of fishmeal was substituted with larval meal, without enzyme complex; LE50−—experimental group where 50% of fishmeal was substituted with larval meal, without enzyme complex; LE60−—experimental group where 60% of fishmeal was substituted with larval meal, without enzyme complex; LC+—control group with enzyme complex; LE40+—experimental group where 40% of fishmeal was substituted with larval meal, with enzyme complex; LE50+—experimental group where 50% of fishmeal was substituted with larval meal, with enzyme complex; LE60+—experimental group where 60% of fishmeal was substituted with larval meal, with enzyme complex.

**Table 3:** Effects of feeding *H. illucens* insect meal and addition of exogenous enzymes to hetero-clarias on growth and production indices.

Specification	PI	SGR%	MGR	WG%
Insect meal effect				
LC−	18.28 ± 6.60 ^a^	1.09 ± 0.07 ^a^	4.51 ± 0.91 ^a^	132.08 ± 33.48 ^a^
LE40−	22.97 ± 8.25 ^bc^	1.31 ± 0.04 ^b^	5.29 ± 1.06 ^b^	169.96 ± 41.67 ^b^
LE50−	21.17 ± 7.40 ^ab^	1.17 ± 0.06 ^ac^	4.97 ± 1.15 ^ab^	154.30 ± 44.10 ^ab^
LE60−	21.03 ± 7.22 ^ab^	1.2 ± 0.07 ^acd^	4.98 ± 0.79 ^ab^	155.27 ± 31.26 ^ab^
Enzyme effect				
LC+	18.02 ± 7.83 ^a^	1.08 ± 0.07 ^a^	4.45 ± 1.03 ^a^	129.40 ± 44.62 ^a^
LE40+	27.00 ± 10.55 ^d^	1.31 ± 0.06 ^b^	5.72 ± 1.58 ^b^	188.42 ± 62.36 ^b^
LE50+	24.58 ± 6.45 ^e^	1.26 ± 0.04 ^bcd^	5.48 ± 0.83 ^b^	174.34 ± 35.13 ^b^
LE60+	23.16 ± 7.40 ^ce^	1.22 ± 0.05 ^bcd^	5.18 ± 0.75 ^ab^	157.63 ± 30.83 ^ab^
*p*-value				
Meal effect	0.002	0.0001	0.001	0.001
Enzyme effect	0.061	0.0009	0.102	0.149
Meal × enzyme	0.621	0.0041	0.612	0.522

Different superscripts among rows in the same column mean significant differences (*p* < 0.05); PI—production index; SGR—specific growth rate; MGR—metabolic growth rate; WG—percentage weight gain; LC−—control group without enzyme complex; LE40−—experimental group where 40% of fishmeal was substituted with larval meal, without enzyme complex; LE50−—experimental group where 50% of fishmeal was substituted with larval meal, without enzyme complex; LE60−—experimental group where 60% of fishmeal was substituted with larval meal, without enzyme complex; LC+—control group with enzyme complex; LE40+—experimental group where 40% of fishmeal was substituted with larval meal, with enzyme complex; LE50+—experimental group where 50% of fishmeal was substituted with larval meal, with enzyme complex; LE60+—experimental group where 60% of fishmeal was substituted with larval meal, with enzyme complex.

**Table 4:** Effects of feeding *H. illucens* insect meal and exogenous enzymes to hetero-clarias on hematological indices and different enzymes.

	Hb (g/dL)	Hct %	Ery (mil/mm^3^)	GPx (U/g Hb)	SOD (U/g Hb)	ALAT (U/L)	ASAT (U/L)
Insect meal effect							
LC−	6.72 ± 0.20 ^a^	34.50 ± 1.19 ^a^	2.55 ± 0.25 ^a^	146.90 ± 2.61 ^bc^	1493.0 ± 6.32 ^a^	8.50 ± 0.55 ^ab^	26.70 ± 0.08 ^a^
LE40−	7.52 ± 0.01 ^a^	33.00 ± 2.37 ^a^	2.46 ± 0.17 ^a^	140.00 ± 0.79 ^ab^	1393.0 ± 17.16 ^bc^	9.95 ± 0.75 ^ab^	31.50 ± 1.26 ^ab^
LE50−	6.33 ± 0.27 ^a^	33.50 ± 0.40 ^a^	3.48 ± 0.30 ^a^	147.50 ± 5.30 ^bc^	1436.0 ± 8.20 ^ab^	11.00 ± 0.87 ^b^	34.48 ± 9.19 ^b^
LE60−	6.85 ± 0.06 ^a^	33.00 ± 2.37 ^a^	2.26 ± 0.03 ^a^	154.00 ± 7.12 ^c^	1568.5 ± 16.50 ^de^	15.10 ± 3.32 ^c^	45.85 ± 0.20 ^c^
Enzyme effect							
LC+	7.55 ± 0.30 ^a^	35.00 ± 1.98 ^a^	2.86 ± 0.02 ^a^	139.95 ± 7.23 ^a^	1407.0 ± 8.70 ^bf^	7.38 ± 0.18 ^a^	25.55 ± 0.75 ^a^
LE40+	7.33 ± 0.08 ^a^	34.00 ± 0.79 ^a^	3.05 ± 0.08 ^a^	133.60 ± 5.06 ^a^	1298.0 ± 7.94 ^g^	8.95 ± 0.20 ^ab^	30.65 ± 2.02 ^ab^
LE50+	7.60 ± 0.46 ^a^	35.00 ± 0.01 ^a^	3.44 ± 0.27 ^a^	144.75 ± 4.94 ^abc^	1356.5 ± 9.39 ^cfg^	10.15 ± 1.38 ^ab^	34.00 ± 3.08 ^b^
LE60+	7.16 ± 0.28 ^a^	34.50 ± 0.40 ^a^	3.36 ± 0.0.07 ^a^	152.65 ± 7.19 ^bc^	1499.5 ± 10.40 ^ad^	14.55 ± 2.65 ^c^	44.35 ± 1.86 ^c^
*p*-value							
Meal effect	0.189	0.138	0.238	0.001	0.001	0.001	0.001
Enzyme effect	0.093	0.597	0.101	0.009	0.001	0.100	0.389
Meal × enzyme	0.078	0.109	0.211	0.126	0.029	0.982	0.990

Different superscripts among rows in the same column show significant differences (*p* < 0.05); Hb—hemoglobin; Hct—hematocrit; Ery—erythrocytes; GPx—glutathione peroxidase; SOD—superoxide dismutase; ALAT—alanine aminotransferase; ASAT—aspartate aminotransferase; LC−—control group without enzyme complex; LE40−—experimental group where 40% of fishmeal was substituted with larval meal, without enzyme complex; LE50−—experimental group where 50% of fishmeal was substituted with larval meal, without enzyme complex; LE60−—experimental group where 60% of fishmeal was substituted with larval meal, without enzyme complex; LC+—control group with enzyme complex; LE40+—experimental group where 40% of fishmeal was substituted with larval meal, with enzyme complex; LE50+—experimental group where 50% of fishmeal was substituted with larval meal, with enzyme complex; LE60+—experimental group where 60% of fishmeal was substituted with larval meal, with enzyme complex.

**Table 5:** Effects of feeding *H. illucens* insect meal and exogenous enzymes to hetero-clarias on serum biochemistry.

Specification	Total Protein (g/dL)	Albumins (g/dL)	Urea (mg/dL)	Creatinine (mg/dL)	γ-Glob (g/dL)	Cholesterol (mg/dL)	Triglycerides (mg/dL)	Total Lipids (mg/dL)
Insect meal effect								
LC−	4.63 ± 0.02 ^a^	1.17 ± 0.08 ^ab^	1.69 ± 0.59 ^a^	0.37 ± 0.05 ^a^	0.02 ± 0.01 ^a^	114.70 ± 2.85 ^ab^	218.70 ± 7.75 ^a^	437.3 ± 32.29 ^ab^
LE40−	3.36 ± 0.17 ^b^	1.12 ± 0.07 ^ab^	1.28 ± 1.86 ^c^	0.32 ± 0.01 ^acd^	0.018 ± 0.01 ^a^	103.05 ± 3.01 ^ae^	210.50 ± 9.37 ^a^	461.0 ± 21.35 ^ab^
LE50−	3.94 ± 0.30 ^de^	1.35 ± 0.01 ^c^	1.62 ± 0.79 ^ab^	0.33 ± 0.02 ^a^	0.014 ± 0.01 ^a^	129.50 ± 6.02 ^b^	298.75 ± 8.86 ^b^	604.7 ± 39.7 ^cd^
LE60−	4.14 ± 0.11 ^e^	1.63 ± 0.13 ^d^	1.69 ± 1.62 ^a^	0.35 ± 0.03 ^ad^	0.015 ± 0.01 ^a^	135.60 ± 5.03 ^b^	359.20 ± 6.41 ^b^	743.2 ± 57.51 ^c^
Enzyme effect								
LC+	2.99 ± 0.19 ^c^	1.11 ± 0.09 ^a^	1.44 ± 1.34 ^bc^	0.25 ± 0.08 ^b^	0.02 ± 0.01 ^a^	109.60 ± 5.08 ^ae^	193.30 ± 7.12 ^a^	377.0 ± 44.27 ^ab^
LE40+	2.90 ± 0.04 ^c^	1.10 ± 0.04 ^ab^	0.83 ± 0.32 ^d^	0.24 ± 0.01 ^b^	0.014 ± 0.01 ^a^	71.35 ± 3.31 ^cd^	205.45 ± 7.27 ^a^	408.1 ± 58.42 ^ab^
LE50+	3.17 ± 0.25 ^bc^	1.27 ± 0.09 ^bc^	1.38 ± 0.83 ^c^	0.26 ± 0.01 ^bc^	0.021 ± 0.01 ^a^	95.05 ± 6.81 ^de^	219.10 ± 7.81 ^a^	506.0 ± 21.35 ^b^
LE60+	3.70 ± 0.13 ^d^	1.49 ± 0.09 ^cd^	1.34 ± 1.03 ^c^	0.24 ± 0.02 ^b^	0.019 ± 0.01 ^a^	95.35 ± 4.72 ^a^	328.45 ± 5.31 ^b^	682.1 ± 30.52 ^cd^
*p*-value								
Meal effect	0.001	0.001	0.001	0.344	0.156	0.001	0.001	0.001
Enzyme effect	0.001	0.009	0.001	0.001	0.087	0.001	0.003	0.001
Meal × enzyme	0.001	0.481	0.147	0.495	0.127	0.017	0.117	0.796

Different superscripts among rows in the same column mean significant differences (*p* < 0.05); γ-glob—γ-globulins; LC−—control group without enzyme complex; LE40−—experimental group where 40% of fishmeal was substituted with larval meal, without enzyme complex; LE50−—experimental group where 50% of fishmeal was substituted with larval meal, without enzyme complex; LE60−—experimental group where 60% of fishmeal was substituted with larval meal, without enzyme complex; LC+—control group with enzyme complex; LE40+—experimental group where 40% of fishmeal was substituted with larval meal, with enzyme complex; LE50+—experimental group where 50% of fishmeal was substituted with larval meal, with enzyme complex; LE60+—experimental group where 60% of fishmeal was substituted with larval meal, with enzyme complex.

**Table 6:** Effects of the administration of *H. illucens* insect meal and exogenous enzymes in the feed of hetero-clarias on the chemical composition of the meat (% of wet matter basis).

Specification	Dry Matter (%)	Crude Protein (%)	Crude Fat (%)	Ash (%)
Insect meal effect				
LC−	20.19 ± 0.04 ^a^	12.61 ± 0.11 ^a^	1.40 ± 0.07 ^ab^	1.09 ± 0.05 ^ab^
LE40−	19.70 ± 0.34 ^a^	12.62 ± 0.14 ^a^	1.54 ± 0.04 ^abc^	1.07 ± 0.07 ^ab^
LE50−	20.63 ± 0.49 ^a^	12.78 ± 0.25 ^a^	1.63 ± 0.25 ^c^	1.01 ± 0.06 ^b^
LE60−	20.44 ± 0.08 ^a^	12.75 ± 0.63 ^a^	1.62 ± 0.02 ^c^	1.09 ± 0.02 ^a^
Enzyme effect				
LC+	20.29 ± 0.28 ^a^	12.74 ± 0.10 ^a^	1.39 ± 0.06 ^a^	1.06 ± 0.02 ^ab^
LE40+	19.84 ± 0.53 ^a^	12.77 ± 0.11 ^a^	1.53 ± 0.07 ^abc^	0.98 ± 0.02 ^bc^
LE50+	20.30 ± 0.18 ^a^	12.86 ± 0.07 ^a^	1.44 ± 0.04 ^abc^	1.06 ± 0.03 ^ab^
LE60+	20.24 ± 0.42 ^a^	12.82 ± 0.63 ^a^	1.64 ± 0.17 ^c^	1.08 ± 0.03 ^ab^
*p*-value				
Meal effect	0.136	0.532	0.001	0.003
Enzyme effect	0.491	0.203	0.219	0.101
Meal × enzyme	0.347	0.984	0.207	0.007

Different superscripts among rows in the same column show significant differences (*p* < 0.05). *p*—*p*-value for bifactorial ANOVA; LC−—control group without enzyme complex; LE40−—experimental group where 40% of fishmeal was substituted with larval meal, without enzyme complex; LE50−—experimental group where 50% of fishmeal was substituted with larval meal, without enzyme complex; LE60−—experimental group where 60% of fishmeal was substituted with larval meal, without enzyme complex; LC+—control group with enzyme complex; LE40+—experimental group where 40% of fishmeal was substituted with larval meal, with enzyme complex; LE50+—experimental group where 50% of fishmeal was substituted with larval meal, with enzyme complex; LE60+—experimental group where 60% of fishmeal was substituted with larval meal, with enzyme complex.

## Data Availability

The data presented in this study are available on request from the corresponding author.

## Abbreviations

The following abbreviations are used in this manuscript:

DM	Dry matter
LM−	Control group without Hostazyme X supplementation
LM+	Control group with Hostazyme X supplementation
LE40−	Experimental group with 40% replacement of fishmeal with *H. illucens* larvae meal without Hostazyme X supplementation
LE40+	Experimental group with 40% replacement of fishmeal with *H. illucens* larvae meal with Hostazyme X supplementation
LE50−	Experimental group with 50% replacement of fishmeal with *H. illucens* larvae meal without Hostazyme X supplementation
LE50+	Experimental group with 50% replacement of fishmeal with *H. illucens* larvae meal with Hostazyme X supplementation
LE60−	Experimental group with 60% replacement of fishmeal with *H. illucens* larvae meal without Hostazyme X supplementation
LE60+	Experimental group with 60% replacement of fishmeal with *H. illucens* larvae meal with Hostazyme X supplementation
ME	Metabolizable energy
SGR	Specific growth rate
FCR	Feed conversion ratio
FI	Daily feed intake
DWG	Daily weight gain
WG	Percentage weight gain
SR	Survival rate
PI	Production index
MGR	Metabolic growth rate
SOD	Superoxide dismutase
GPx	Glutathione peroxidase
ALAT	Alanine aminotransferase
ASAT	Aspartate aminotransferase
